# Push-Out Bond Strength of Resin-Modified Glass Ionomer Cement and Flowable Composite Luting Systems on Glass Fiber Post of Root Canal

**DOI:** 10.3390/ma14226908

**Published:** 2021-11-16

**Authors:** Jalison Jacob Cheruvathoor, Lincy Rachel Thomas, Lirin Ann Thomas, Madhuniranjanswamy Mahalakshmamma Shivanna, Pramod Machani, Sachin Naik, Abdulaziz Abdullah Al Kheraif

**Affiliations:** 1 Penang International Dental College, Butterworth 12000, Penang, Malaysia; jalisonjacob@gmail.com (J.J.C.); dr.madhuniranjanswamy@gmail.com (M.M.S.); 2 Asian Institute of Medicine, Science and Technology University, Semeling, Bedong 08100, Kedah, Malaysia; raclin88@gmail.com (L.R.T.); liann88@gmail.com (L.A.T.); 3 Department of Prosthodontics, Faculty of Dentistry, Lincoln University, Jalan Stadium SS 7/15, Ss 7, Petaling Jaya 47301, Selangor, Malaysia; ppmachani@gmail.com; 4 Dental Biomaterials Research Chair, Dental Health Department, College of Applied Medical Sciences, King Saud University, Riyadh 11433, Saudi Arabia; aalkhuraif@ksu.edu.sa

**Keywords:** flowable composite, glass fiber post, luting cement, resin-modified glass ionomer cement, root canal

## Abstract

Background: Posts that have been properly fitted can withstand torsion forces and so provide better retention. The push-out bonding strength of glass fiber posts to the root canal was evaluated using resin-modified glass ionomer cement (RMGIC) and flowable composite (FC). Method: Forty single-rooted maxillary central incisors were used in the study. The samples were randomly divided into two groups of 20 teeth each. The crown-down procedure was used to clean and shape the pulp area. A Tenax fiber trans Coltene whaletene post was used by both groups. The first group utilized FC (Filtek Z 350 3M ESPE) to coat the post, whereas the second group used RMGIC (Rely X 3M ESPE). The specimens were cross-sectioned after 24 h. Specimens were cross-sectioned four millimeters thick into coronal and middle parts using a sectioning machine, yielding 40 specimens per group. The strength of the bond between the luting cement and the posts was measured using push-out bond strength testing. We loaded the components at a cross speed of 0.5 mm/min on a universal testing machine until the bond failed. Results: The FC group had a 4.80 N push-out bond strength, whereas the RMGIC group had a 7.11 N push-out bond strength. Conclusion: FC’s mean push-out bond strength score is lower than RMGIC’s.

## 1. Introduction

The difficulty of restoring teeth that are endodontically treated has led to a wide range of base restorations. Loss of tooth structure due to endodontic access preparations, caries, and defective restorations makes restoring a pulpless tooth challenging [1]. The length of a post, diameter, design, canal shape and preparation, luting agent, cementation procedure, and other factors all influence post retention [2,3]. A good adaptation of the post to the root canal (RC) will remain for a long time and tolerate torsion forces. The resin adhesives in well-fitting canals provide good retention [4]. The quality of luting cement is the main element in retention. Characteristics such as easy manipulation, a thinner film, extended working time with a fast set, and low solubility are ideal properties in luting cement [5]. However, there have been fewer studies that provide evidence on the comparison of the bond strength of luting cement. Most studies have conflicting results on which of the luting types of cement have superior bond strength [6,7,8].

Based on the unpredictable results of previous studies, bonding RC posts with resin cements, conventional glass ionomer cement, or resin-modified glass ionomer cement (RMGIC) is recommended for luting fiber posts [9,10]. Unlike conventional GICs, RMGIC presents low sensitivity to moisture and strong bonding to tooth structure. It also releases fluoride and shows high compression resistance compared to zinc phosphate cement [6,11]. A new modified hybrid resin material was developed recently to overcome the polymerization shrinkage associated with classic composite resin. In 1996, flowable composite (FC) with a lower filler load was introduced [12]. These materials improved flow, effectively decreased the modulus of elasticity, reduced micro-leakage by enhancing adaptability and this property generates a stress-absorbing layer [13]. As a result, gap formation at the flowable resin and tooth boundary was reduced. Micromechanical retention and chemical interaction are required for the adhesion between monomer acidic groups and hydroxyapatite [14,15,16]. Thus, we conducted the present study to evaluate the push-out bond strength of glass fiber posts to the RC using resin-modified glass ionomer cement and flowable composite.

## 2. Materials and Methods

The study was conducted in Vinayaka Mission’s Sankarachariyar Dental College (VSDC) and Sree Chitra Tirunal Institute for Medical Science and Technology, Biomedical Technology Wing, Poojappura, Thiruvananthapuram. Ethical approval was obtained from VSDC.

**Figure 1:**
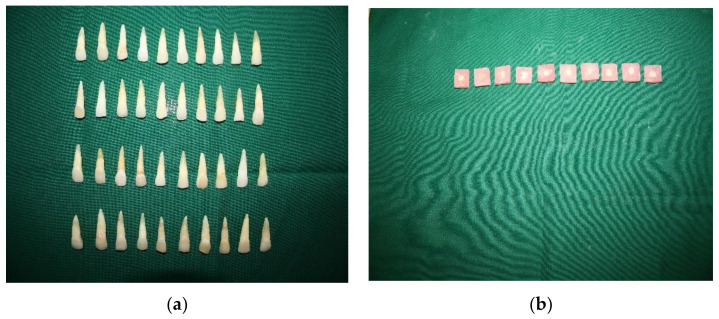
(**a**,**b**) Natural single-rooted maxillary central incisor teeth and mounted molds cross-sectioned 4 mm thick into coronal and middle parts.

A total of 40 extracted natural single-rooted maxillary central incisor teeth were used in the study. Selected teeth were extracted due to periodontal problems and the structure of the tooth, i.e., with enamel and dentine intact, so that results can be generalized. At the cementoenamel junction, teeth were sectioned with a high-speed airotor handpiece. The working length was determined by cutting 0.5 mm short of the apex using a #10 K flex file. The pulp space was cleaned and shaped using the crown-down technique with a rotary pro-taper nickel-titanium file up to size F2. Irrigation with sodium hypochlorite 5.25% solution, saline, and 17% Ethylenediaminetetraacetic acid (EDTA) was done at the same time. The RCs were dried with paper points after being irrigated with distilled water. All of the RCs were sealed with a calcium hydroxide-based sealer (apexit plus), and then gutta-percha was used to obturate them. The teeth were then stored in deionized water for 24 h to create post spaces. The RC walls of the specimen were individually enlarged using low-speed drill tips. The depth of the post space was 9 mm. The specimens were divided into 2 groups of 20 samples each at random (Figure 1a).

Group 1: The RCs were etched for 10s with 37% phosphoric acid, then rinsed with water and dried. A micro brush was used to apply the single-bond universal adhesive to the RCs. After that, the post (Tenax fiber trans Coltene whaletene) was coated with FC (Filtek Z 350 3M ESPE) and put into the RC, with the excess resin later removed. Then the components were light cured for 60 s.

Group 2: The RC wall was etched for 10 s with 37% phosphoric acid, then rinsed with a water syringe and dried with a paper tip. The post was put in the RC and covered with RMGIC (Rely X 3M ESPE), and the post was light cured for 60s.

Plastic molds were used to mount all of the samples on epoxy resin, which was allowed to set for 24 h, and all specimens were cross-sectioned 4 mm thick into the coronal and middle parts by a sectioning machine yielding 40 specimens per group (Figure 1b).

**Figure 2:**
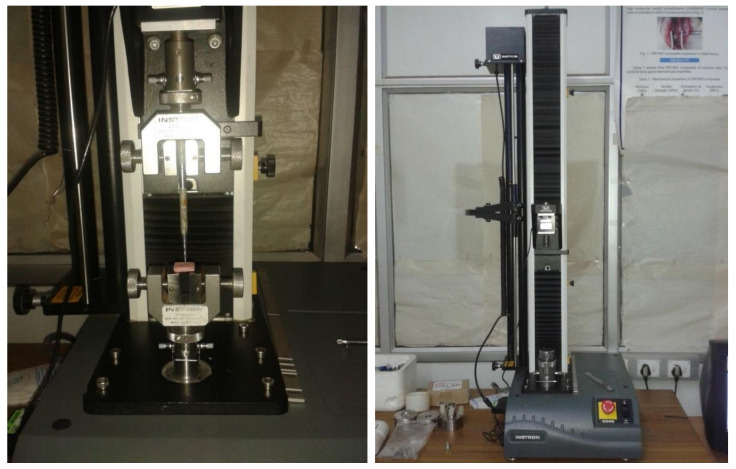
Testing machine to assess push-out bond strength.

The push test was used to calculate the bond strength between the luting cement and post. On the testing machine, the post was loaded with a 1 mm in diameter cylindrical plunger (Figure 2).

The plunger point was positioned to touch only the post, leaving the surrounding post space walls. Loading was done on a universal testing machine (Instron universal testing machine, USA) at 0.5 mm/min crosshead speed until bond breakdown occurred. To debond the recorded post, force (N) is necessary. The following formula was used to calculate the binding strength in MPa:

N/2·πrh

where,

π = 3.14
r = post radius,
h = thickness of the slice in mm.

The data were analyzed using unpaired Student’s *t*-test *p*, and the scores tested for a significant difference.

## 3. Results

The difference between the mean scores of the push-out bond strength value was tested between two groups, namely RMGIC and FC, using the unpaired Student’s t-test. Date verified by the Kolmogorov–Smirnov test and data distribution were normal (Figure 2).

**Figure 3:**
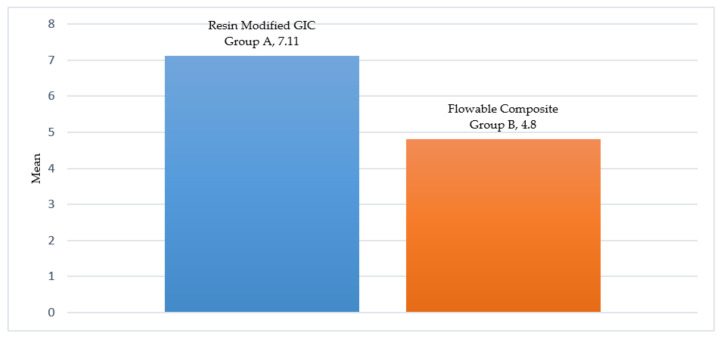
“Push-Out “bond strength of groups A and B.

**Figure 4:**
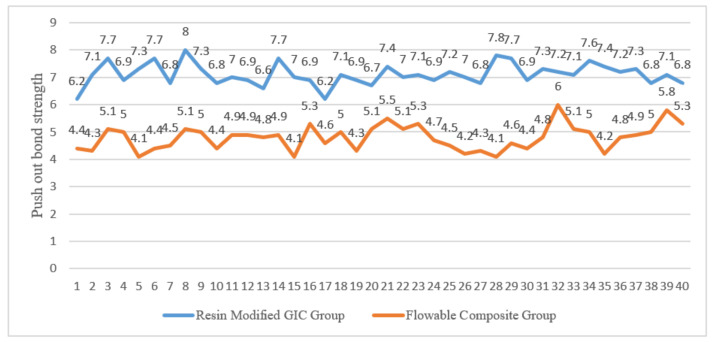
Individual “Push-Out “bond strength of each sample.

Table 1 shows the sample descriptive statistics and the unpaired Student’s t statistics. There is a very significant difference in the mean push-out bond strength score observed when the *p*-value is less than 0.01. Figure 3 and Figure 4 show the mean push-out bond strength score of the FC group and RMGIC group.

## 4. Discussion

According to previous literature, RMGIC has shown increased physical properties, such as less sensitivity to moisture, high dimensional stability, chemical, and micromechanical bonding, higher bonding to the tooth, enhanced adaptation to canal walls, hybrid layer formation, and hygroscopic expansion leading to increased frictional resistance [17,18,19,20,21].

The type of RC also affects retention, as Maryam et al. found that the narrow channel held water due to surface tension, making it difficult for the bonding agent to replace it. As a result, even though the RCs were dried using paper points, the bond strength of the etching and rinse technique was lowered due to increased moisture content [22]. Ferrari et al. also established that different sections of the same RC did not respond to acid etching in the same way, and as a result, dentine bonding abilities vary at different depths inside the same RC [23].

The hydrophilic monomers in the self-etching adhesives are more concentrated. The water content in the solvent affects the combination of adhesive and light-cured composite. Suh BI et al. reported that a high concentration of acidic monomers in adhesive systems reduced the rate of polymerization for light-curing composites [24].

The degree of friction, rather than bonding to the RC, was found to be more reliable in determining fiber post displacement in a recent study. Due to its hygroscopic expansion property RMGIC can be utilized for fiber post luting. The post dislodgment frictional resistance is increased by the hygroscopic expansion [18].

In our study, RMGIC (7.11 N) achieved the best bond results. The study conducted by Maryam et al. concluded that conventional glass ionomer cement achieved the best bond results. Water content in the cement will lead to a reduction in time to interact with the substrate. Several studies reported contradicting results when GIC was used as a bonding agent. This may be because of the usage of 40% citric acid and 3% NaOCl before cementation [22]. The reaction of RMGIC results in an enhanced adaptation to the RC, which may favor the friction resistance to dislodgment of the post. An explanation would be that the RMGIC benefits from hygroscopic expansion, leading to an increased frictional strength [21]. Bonfante et al. examined the bonding effect of RMGIC to glass fiber posts. They concluded RMGIC forms a hybrid layer between dentine and found the best option [25].

FC in vitro abrasion wear experiments have yielded mixed results, and the clinical wear resistance of FC is still unknown. Due to the low fillers composition and physical properties, flowable materials should only be used in low-stress environments. Compared to conventional mini-filled hybrids, which have a filler loading of 50% to 70%, light-cured FC has a filler loading of 37% to 53% [26,27]. Bayne et al., in a study conducted to evaluate the mechanical properties of FC, concluded that in high-stress bearing areas, flowable materials should be utilized with caution [28].

However, FC still has the advantages of a high wet ability ensuring penetration into every irregularity; the ability to produce thin layers, hence reducing or eliminating air inclusion or entrapment; and, because of their high flexibility, they are less prone to be displaced in high-stress regions [26].

According to Mojon et al., RMGIC is an ideal luting agent for patients with a high caries index due to its fluoride release and better adaptation to RC [25]. When comparing the push-out bond strength test to the conventional shear bond strength test, the push-out bond strength test provides a superior measure of bonding effectiveness. Roydhouse (1970) was the first to advocate for the push-out test in dentistry [29].

According to reports, when push-out tests were done on the entire post or thick root portions using a thin-slice specimen, nonuniform stress developed at the adhesive barrier. The thin-slice push-out test allowed for a more uniform stress distribution along with the bonded interface. As a result, we adopted a push-out design with 4 mm sections in our work, similar to Bitter et al., Farina et al., and Kremeier et al. [22]. Shear stress is created at the post-cement interface and the dentin-cement interface during push-out testing. The stresses used in clinical conditions are more similar to this than the linear shear test. Furthermore, Goracci et al. found that the push-out test is more efficient and reliable than the micro-tensile approach in a study [30].

Over time, luting cement has developed into stronger materials that are easier to use and can bond teeth effectively. Concurrently, luting materials have also developed, offering a wider variety of materials and far more aesthetic solutions. With the wide range of cement materials and newer emergent materials, it is critical to select a luting cement that meets the requirements of the mutually intraoral environment and the type of restoration.

The study’s limitations include the fact that it was conducted in vitro, which may not fully replicate oral environments. Furthermore, we used a single load to assess bond strength.

## 5. Conclusions

We conclude that resin-modified glass ionomer cement (RMGIC) has a more resistant mean push-out bond strength score than flowable composite (FC). It is critical to preserve teeth by cutting into them as little as possible during the preparation. Internal dentin preservation should thus be a primary goal of both endodontic therapy and future restorative procedures. FC has a variable composition, and as a result, the material has a wide spectrum of mechanical and physical properties. In order to select the appropriate materials, clinicians must be aware of its indications to a specific clinical situation.

## Figures and Tables

**Table 1:** Descriptive statistics.

Group	*n*	Mean	Std. Deviation	*t*	*p*
Resin-Modified GIC Group (A)	40	7.11	0.396	24.15	<0.001 **
Flowable Composite Group (B)	40	4.80	0.459		

## Data Availability

Data are contained within the article.
